# Formation of Polycyclic Aromatic Hydrocarbons on Grilled Pork Neck Loins as Affected by Different Marinades and Grill Types

**DOI:** 10.3390/foods14101673

**Published:** 2025-05-09

**Authors:** Marta Ciecierska, Urszula Komorowska, Marcin Bryła, Marek Roszko

**Affiliations:** 1 Department of Food Technology and Evaluation, Institute of Food Sciences, Warsaw University of Life Sciences, Nowoursynowska 159 Street, 02-787 Warsaw, Poland; ula.komorowska@o2.pl; 2 Department of Food Safety and Chemical Analysis, Prof. Waclaw Dąbrowski Institute of Agricultural and Food Biotechnology-State Research Institute, 02-532 Warsaw, Poland; marcin.bryla@ibprs.pl (M.B.); marek.roszko@ibprs.pl (M.R.)

**Keywords:** PAHs, pork neck loins, grilling, marinades, QuEChERS, HPLC–FLD/DAD, GC/MS

## Abstract

Processing methods affect the quality and, most importantly, safety of meat. The effects of various marinades, a kind of green processing technology commonly used in Poland, on PAH contamination in pork neck loins, the most frequently grilled pork meat, were investigated, including universal, pork, and honey mustard, as well as the most popular grilling tools. It is important to note that no such data have been published so far. Our previous study focused on poultry meat, another commonly grilled meat. PAH analysis was conducted using the QuEChERS–HPLC–FLD/DAD method and confirmed by the GC/MS method. Weight loss and changes in individual color parameters after grilling were also analyzed. Grilling on a charcoal grill without an aluminum tray caused statistically the greatest PAH contents. Some of these samples, according to Commission Regulation (EU) No. 915/2023 restrictions, should not be consumed by humans due to the high content of B[a]P (5.26–6.51 µg/kg). The lowest contamination levels overall were determined for the ceramic contact grill. Studies have also shown that the universal and pork marinades can reduce PAH contamination by about 24–29% for 4 heavy PAHs and by 31–32% for 15 PAHs, whereas the honey mustard marinade increases their accumulation in grilled products by 13% for 4 PAHs and 12% for 15 PAHs. Carefully choosing the grilling equipment, such as using electric grills instead of charcoal or using aluminum trays when grilling with charcoal and marinating the meat before grilling, is essential for food producers and consumers. These practices can significantly reduce the harmful health effects of PAHs, making them vital steps toward safer food preparation.

## 1. Introduction

Polycyclic aromatic hydrocarbons (PAHs or polyarenes) are a diverse group of colorless, white, pale yellow, or green solids belonging to the category of organic compounds, consisting of carbon and hydrogen atoms joined by benzene ring systems. Depending on the number of benzene rings in the molecule, polyarenes are divided into light PAHs (depending on classification, 2–3 or 2–4 rings) and heavy PAHs (4–5 or more rings). In addition to carbon and hydrogen, polyarenes can contain nitrogen, sulfur, or oxygen atoms attached to the benzene rings [1,2].

Over the years, many organizations have studied the effects of polyarenes on animal and human organisms to monitor the harmful effects of polycyclic aromatic hydrocarbons [3]. The origins date back to 1976, when the United States Environmental Protection Agency [4] set a 16-PAH priority list, including the most known benzo[a]pyrene (B[a]P), as indicator compounds with strong to weak toxicity for humans. In December 2002, the Scientific Committee on Food of the European Union [5] classified 15 heavy PAHs as the most dangerous for humans. Three years later, the FAO/WHO Expert Committee on Food Additives [6] identified 13 of these as mutagenic and carcinogenic. Commission Recommendation (EC) No. 108/2005 and the EFSA Panel on Contaminants in the Food Chain (CONTAM) stated that B[a]P is not a good indicator of other PAHs in foodstuffs [7]. Consequently, the sum of four heavy polyarenes—benzo[a]pyrene, benzo[a]anthracene (B[a]A), benzo[b]fluoranthene (B[b]F), and chrysene (Chr)—has been recognized as the most reliable marker for the presence of PAHs in foodstuffs [8].

Apart from the natural environment, the main route of exposure to polyarenes is the consumption of food, in particular processed food. Meat and animal products are the second-largest food product consumption group, and they pose a risk of dietary PAH intake. The type of meat, the content of fat in which contaminants accumulate, and the food processing method greatly impact PAH contamination. In particular, heat-treated products are exposed to PAH formation. The kind of meat, the fat content, the method and time of heat processing, and the heat source significantly affect PAH concentration in such products [9,10,11].

Due to the high risk of PAH contamination in processed food products, selecting the appropriate raw material and the heat treatment method can effectively minimize the danger of these compounds [12]. In the case of grilling, which is a popular method of food preparation, especially in the summer season, two methods of meat preparation, direct and indirect grilling, can be distinguished. In the case of both methods, the raw material is placed over the heat source, with the difference being that in the indirect method of grilling, the meat is not placed directly over the heat source [13,14]. Preparing food using a tray or aluminum foil minimizes contact between the melting fat and the heat source, and less PAH is produced [15]. Electric and ceramic grills, used in homes and restaurants, are also becoming increasingly popular. It is also important to properly prepare the raw material before it is cooked. Marinating food not only enables one to give dishes a unique taste and smell, but some studies have shown that it has beneficial health effects, inhibiting the formation of harmful compounds such as PAHs [16,17].

Commission Regulation (EU) No. 915/2023 regulates the permissible amount of polyarenes in food products subjected to grilling. It sets the maximum B[a]P concentration at 5 μg/kg and the content of the four heavy PAHs at 30 μg/kg [18].

Studies analyzing the formation and occurrence of PAHs in grilled meat products, as well as developing strategies to prevent and reduce PAH contamination, are still crucial. Although numerous researchers have focused on minimizing PAH levels in heat-treated meats, they typically emphasize the 16 EPA-listed PAHs. Nevertheless, in line with the recommendations issued by the EFSA and SCF and discussed earlier, scientific investigations must target the more toxic polyarenes from the SCF list. It is also worth noting that a limited number of studies have indicated that marinating in addition to reducing PAH contamination may also result in a higher increase in PAHs compared to grilled meat that has not been marinated. Considering the above, this study examined PAH contamination in pork neck loins marinated with formulations commonly used in Poland and heat-treated with charcoal and electric grills. It should be emphasized that no such data have been published so far. Our previous study examined poultry meat, another frequently grilled meat in Poland. The research objectives are to select the marination and grilling tools whose use minimizes PAH contamination in pork neck loins and enhances the safety of such products, and conversely, which grills and marinades are conducive to a higher level of contamination, and to suggest effective strategies for reducing these harmful compounds to minimize their intake via diet and promote safer food consumption. PAH analysis was devoted to 19 PAHs, including 15 heavy PAHs from the SCF list, B[a]P, 4 heavy marker PAHs, and 4 light PAHs from the EPA list, which are generally the most prevalent in PAH contamination profiles. Polyarene determination was conducted with the QuEChERS extraction method, an up-to-date and streamlined sample preparation method, avoiding complicated and time-consuming analytical steps with liquid chromatography and fluorescence and diode array detectors (QuEChERS-HPLC-FLD/DAD), and was confirmed by gas chromatography coupled with mass spectrometry (GC/MS). The findings of this research will offer crucial insights into how grilling methods and marination influence PAH contamination levels in grilled pork meat.

## 2. Materials and Methods

### 2.1. Pork Type, Sample Preparation for Grilling, and Experimental Design

The research material was pork neck loins from a Warsaw market in Poland. The samples were portioned into 100 g and subsequently underwent a marination process using preselected marinades commonly used in Poland for pork neck marination treatment: the universal, pork, and honey mustard marinades. These marinades were deliberately chosen due to their widespread consumer preference. Each marinade was prepared strictly as stated in the manufacturer’s instructions, with their exact formulations detailed in Table 1.

The unmarinated pork neck loins and the samples after marination treatment were placed in a refrigerator (4 ± 1 °C) in glass food containers until the next day. Each sample was prepared for three repetitions. The grilling was conducted twice (n = 6). The next morning, prior to heat treatment, all pork neck loins were taken out of the refrigerator and rested until their internal temperature stabilized to between 15 and 20 °C.

The procedure of the experiment was designed to encompass marination with preselected marinades, heat treatment on various grill types, the measurement of weight loss post grilling, the assessment of color parameters in both unprocessed and grilled samples, preparation for PAH analysis via the QuEChERS method, and comprehensive qualitative and quantitative analysis using high-performance liquid chromatography with fluorescence and diode-array detectors (HPLC–FLD/DAD), and the results confirmation by gas chromatography coupled with mass spectrometry (GC/MS).

### 2.2. Grill Types and Parameters of Grilling

To evaluate the influence of grilling methods and grill types on PAH formation, the following grills, which had previously been described by Ciecierska and Komorowska [14], were utilized: a charcoal grill (Weber Original Kettle E-4710, Weber-Stephen Deutschland GmbH, Ingelheim am Rhein, Germany) without a tray and with an aluminum tray, an electric cast iron contact grill with two corrugated surfaces (Combi Grill, GR-1000 model, Optimum, Mińsk Mazowiecki, Poland), and an electric ceramic contact grill with a corrugated and smooth surface for the top and bottom, respectively (SpidoCook, XP010PR model, UNOX, Cadoneghe, Italy).

Before heat treatment, all grills were preheated for a minimum of 10 min. During this period, the initial weights of the pork neck loins were noted. Once the preheating was complete, the grilling process started. The specific grilling parameters for each grill are detailed in Table 2. Upon reaching the target medium doneness and the required internal temperature at the geometric center of the samples (>80 °C), they were immediately taken from the grill, cooled, and re-weighed, and subsequently subjected to PAH determination.

### 2.3. Weight Loss

To assess the weight loss, the pork neck loins were weighed in three repetitions, both prior to and post grilling. The formula for evaluating the cooking loss of the heat-treated meats is as follows:

$$Weight\;loss\left(\%\right)=\frac{M-M_{g}}{M}\cdot 100$$

M—the weight of the unprocessed pork neck loins.

M_g_—the weight of the grilled pork neck loins.

### 2.4. Color Measurement

The CIE L*a*b* color parameters of both the unprocessed and grilled meat samples were measured using a Minolta Colorimeter (Chroma Meter CR–200, Konica Minolta Corp., Japan), calibrated with a white reference plate (L* = 97.83, a* = −0.45, b* = +1.88). The color differences between the samples prior to and post grilling (ΔE) were determined using the measured lightness (L*), redness (a*), and yellowness (b*) values.

∆E = [(∆L)^2^ +(∆a)^2^ +(∆b)^2^]^0.5^

### 2.5. Chemicals and Materials

The acetonitrile (of HPLC gradient grade), sodium chloride, and anhydrous magnesium sulfate (analytical purity > 99.0%) were procured from Avantor Performance Materials Poland S.A. (Gliwice, Poland). The sorbents utilized in the QuEChERS method included primary secondary amine (PSA), silica gel modified with C18 groups, and graphitized carbon black (GCB), specifically Sepra PSA Bulk Packing, Sepra C18–E Bulk Packing, and Sepra GCB Bulk Packing, obtained from Phenomenex (Warsaw, Poland). Standard mixtures of 15 SCF PAHs (PAH–Mix 183, Dr. Ehrenstorfer, Augsburg, Germany) and 16 PAHs from the US EPA list (PAH–Mix 9, Dr. Ehrenstorfer) were obtained from Witko (Łódź, Poland), together with a deuterated PAH mixture (PAH-Mix 918, Dr. Ehrenstorfer) used for the quantitative confirmation of results by the GC/MS method. The 15 SCF PAH mixture consisted of benzo[a]pyrene, benzo[a]anthracene, benzo[b]fluoranthene, chrysene, 5–methylchrysene (5-MChr), cyclopenta[c,d]pyrene (C[cd]P), benzo[j]fluoranthene (B[j]F), benzo[k]fluoranthene (B[k]F), benzo[g,h,i]perylene (B[ghi]P), indeno[cd]pyrene (I[cd]P), dibenzo[a,h]anthracene (D[ah]A), dibenzo[a,l]pyrene (D[al]P), dibenzo[a,e]pyrene (D[ae]P), dibenzo[a,h]pyrene (D[ah]P), and dibenzo[a,i]pyrene (D[ai]P). The second PAH standard mixture was exclusively used for the quantification of the four light PAHs: phenanthrene (Phen), anthracene (Anthr), fluoranthene (F), and pyrene (Pyr). Deionized water was obtained from a Millipore Milli-Q water purification system. Polytetrafluoroethylene (PTFE) syringe filters (25 mm i.d., 1 µm pore size) and Falcon centrifuge tubes (PTFE) were sourced from BioAnalytic (Gdańsk, Poland).

### 2.6. PAH Determination with the QuEChERS–HPLC–FLD/DAD Method

The preparation of samples and PAH determination were conducted using the methodology outlined by Ciecierska and Komorowska [14] in a previous scientific work on other research material and Ciecierska et al. [19]. The methodology consisted of PAH extraction and sample purification using the QuEChERS method, followed by subsequent chromatographic analysis using the HPLC–FLD/DAD system, and results confirmation by the GC/MS technique.

Briefly, 5 g of previously homogenized pork neck loins was weighed into a 50 mL Falcon tube and vortexed for 1 min with 10 mL of acetonitrile to extract fat and PAHs. Subsequently, 4 g of MgSO_4_ and 1 g of NaCl were added, and the Falcon tube content was vortexed (3 min) and then centrifuged (3400 rpm, 3 min, using a laboratory centrifuge MPW—352R, Warsaw, Poland). Following phase separation, the organic layer (4 mL) was applied into Falcon 15 mL tubes with purification sorbent material (MgSO_4_, PSA, C18, GCB), then shaken on a vortex (3 min) and subjected to centrifugation (3400 rpm, 3 min). The supernatant after filtration (PTFE syringe filters with 0.2 µm pore diam.) to a chromatographic vial was subsequently analyzed using an HPLC–FLD/DAD system and after changing the solvent to cyclohexane, analyzed by GC/MS to confirm the results.

The determinations of 4 light and 15 marker and heavy SCF PAHs in the pork neck loins were conducted following the methodology described by Ciecierska and Komorowska [14]. A Nexera Shimadzu LC–40 DXR system (Kyoto, Japan), which consisted of FLD and DAD detectors (respectively, RF–20XL and SPD–M10A vp), was utilized. Chromatographic separations were carried out using Kinetex 3.5 µm PAH LC Column 150 × 4.6 mm at 30 °C with specific gradient elution conditions, employing a mobile phase composed of ACN and H_2_O with a 1.5 mL/min flow rate. Fluorescence and diode-array detection parameters varied by compound, and they were presented in the abovementioned methodology [14].

### 2.7. Quantitative Analysis and Validation

**Figure 1:**
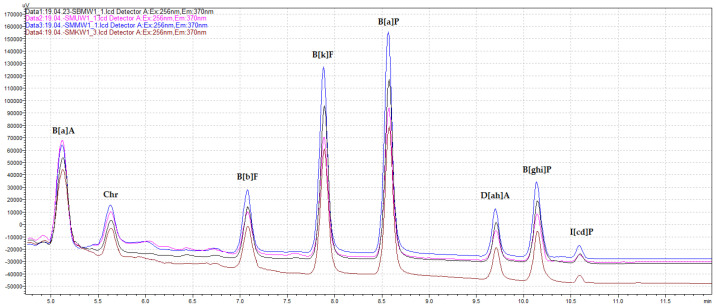
The heavy SCF-PAH chromatogram from the HPLC–FLD technique for pork neck loins in universal (pink), honey mustard (blue), and pork marinades (brown), and unmarinated (black); heat-treated using the charcoal grill with no tray.

The quantification and validation of polyarene determination using the QuEChERS–HPLC–FLD/DAD technique for the grilled meat samples were characterized in a previous study conducted by Ciecierska and Komorowska [14]. The validation parameters for the 4 heavy marker PAHs, including the LOD (0.06–0.08), LOQ (0.12–0.16), recovery rates (%) with relative standard deviations (84.8 ± 7.7%–89.2 ± 7.7%, and HORRAT_R_ values (0.6–0.7), confirmed compliance with Commission Regulation (EU) No. 836/2011 [20]. Additionally, the method exhibited satisfactory performance for both the other SCF-PAHs and the 4 light EPA-PAHs [14]. Figure 1 presents the heavy polyarenes detected in the pork neck loin samples, both after different marination treatments and without marination, grilled using the charcoal grill with no tray.

### 2.8. Confirmation of Results by GC/MS Method

The samples were also analyzed by gas chromatography with mass spectrometry (GC/MS) using an Agilent 7890A/5975C VL MSD Gas Chromatograph-Mass Spectrometer (Agilent Technologies Inc., Santa Clara, CA, USA) to confirm the results obtained by the HPLC-FLD/DAD method. This confirmation was performed using the standard deuterated PAH mixture mentioned earlier. The following conditions were utilized: GC Agilent J&W Select PAH Column (30 m × 0.25 mm × 0.15 µm), a helium carrier gas flow of 1.2 mL min^−1^, an inlet temperature of 300 °C, an injection volume of 1 μL with the splitless injection mode; the MS Quad and MS source temperature of 200 and 300 °C, respectively. The temperature program was as follows: 40 °C (2 min), 40–180 °C (15 °C/min), 180 °C (1 min), 180–200 °C (2 °C/min), 200 °C (1 min), 200–320 °C (3 °C/min), and 320 °C (15 min). The ion abundance within the *m*/*z* range of 100–400 was measured using a detector voltage of 1.5 kV and electron ionization at 70 eV. The selected ion monitoring (SIM) mode was employed for each PAH, measuring the two most abundant and characteristic ions. The identification of PAHs was based on comparing particular analyte retention times in real samples with those of standard PAH solutions, along with the characteristic ions monitored for each compound. For the tested samples of grilled pork neck loins, the data obtained from the GC/MS and HPLC–FLD/DAD methods demonstrated statistically insignificant differences, confirming the reliability of the applied analytical approach.

### 2.9. Statistical Analysis

Statistical analysis was conducted using Statistica v.10-PL (Stat Soft Inc., Tulsa, OK, USA). The data distribution normality was evaluated by comparing *p*-values, with the null hypothesis rejected at *p* ≤ 0.05. To test the significance of differences in mean polyarene contents across all types of grilled pork neck samples regarding the type of grill and the marinades used, an ANOVA analysis and Tukey’s test were applied (α = 0.05). Furthermore, the Pearson correlation was analyzed to examine relationships between weight loss, color components, and the concentrations of the 15 SCF PAHs and all 19 polyarenes.

## 3. Results and Discussion

### 3.1. Meat Weight Loss Analysis After Grilling

The process of grilling melts fat, evaporates water, or leaks cell juices from the raw material, thus reducing its weight after heat treatment [21]. The weight loss was within the 30.0–54.8% range (Table 3), depending on the grilling method and tool chosen and the sample type. The greatest meat weight loss was found using the charcoal grill with no tray and the cast iron electric contact grill (46.6–54.8%), and the lowest was found on the ceramic contact grill (30.0–43.4%). The Pearson analysis showed no correlation between weight loss and contamination with the 15 heavy PAHs (*p* = 0.119). The lack of a linear correlation may be because the weight loss of individual samples was influenced by factors such as different types of grills, temperature, and the duration of grilling. This greatly impacted the slope of the regression line and, thus, the correlation rate. Furthermore, analysis of the correlation between weight loss and the total concentration of the 19 PAHs revealed only a weak positive correlation (*p* = 0.027), which may result from the formation of a relatively large amount of light PAHs in the process of fat pyrolysis.

Using the charcoal grill with no tray and the highest grilling temperature caused one of the greatest instances of weight loss. The aluminum tray reduced the direct contact of the raw material with the heat source and significantly reduced the weight loss. The difference was also observed for electric grills, where a cast iron grill showed a much greater weight loss than a ceramic grill. The results agreed with the conclusion formulated by Purslow, Oiseth, Hughes, and Warner [22] that during cooking, water is expelled from the meat, which causes its weight to decrease. The same researchers concluded that the higher the grilling temperature, the greater the weight loss. Latoch, Głuchowski, and Czarniecka–Skubina [23], Alugwu, Okonkwo, and Ngadi [24], and War Nur Zahidah et al. [25] also showed that with higher temperatures of cooking as well as longer cooking times, weight loss was significantly greater.

### 3.2. Color of Pork Neck Loins

Table 4 depicts the impact of various marinades and types of grills on the color parameters of pork neck loins. The obtained results indicate that the color parameters of the samples were affected by the different marinades and cooking methods. By analyzing the parameter L*, it was noted that the sample marinated in the honey mustard marinade and processed on the charcoal grill with no tray was significantly brighter (48.46), and the unmarinated sample was significantly darker (55.88) than the other samples. Compared to the electric grills on both the cast iron and ceramic grills, significantly higher values of parameter L* were obtained for both samples—marinated in honey mustard marinade and unmarinated. For the a* color component, the highest redness value was shown in the unmarinated charcoal grill sample with no tray (12.21) and in the honey mustard marinade sample from the same grill but with an aluminum tray (11.01). However, there were no statistically significant differences between the different types of marinade for the electric grills. In the case of parameter b*, the value of the yellow component depends on the tested variant. The highest proportion of yellow was noted in meat marinated in the universal marinade and grilled on charcoal grills (SMUW1—14.01 and SMUW2—14.45), while the lowest was recorded for samples without a marinade and prepared on electric grills (SBME2—11.76 and SBME1—13.51).

Comparing changes in the analyzed samples’ color parameters prior to and post grilling, the ΔE parameter was evaluated and is shown in Table 5. In some samples (SMKW1, SMKE1, SMME1, and SMUE2), the color differences due to the heat treatment applied were invisible to an inexperienced person. A slight color change in the universal and the pork marinade could be explained by the intense color of the spice and, in the case of the honey mustard marinade, by the lowest grilling temperature and the low pressure of the heating plates on the product in samples from the ceramic contact grill.

A Pearson correlation analysis between color parameters and PAH concentrations in pork neck loins was carried out. The analysis revealed a weak negative correlation (*p* = 0.008) between the L* parameter and the sum of 19 polyarenes, as well as a weak positive correlation between the a* parameter and the sum of the content of 15 PAHs (*p* = 0.021) and 19 PAHs (*p* = 0.019). No significant correlation was observed between lightness and the total 15-PAH content, nor between yellowness and the total 15-PAH and 19-PAH contents.

Based on the study results, marination treatments and grilling techniques significantly impact the color of the tested pork neck loins. Furthermore, the Pearson correlation analysis proves that a lower proportion of redness (a*) corresponds to a higher PAH contamination of the samples. In contrast, lighter colored samples exhibit lower light and heavy PAH content levels. The findings agreed with studies conducted by other researchers. Assogba et al. [26], in their research, proved that pork, after grilling, becomes darker due to such factors as the Maillard reaction, the pyrolysis of fat, the carbonization of proteins, the caramelization of sugars, or the denaturation of hemoglobin and myoglobin. Moreover, Cho et al. [27] confirmed that marination influences the color parameters of barbecued pork patties. Silva, Hamer, and Guénard [28] reported that the proportion of redness and yellowness increases due to the grilling process. In addition, the research conducted by Wang, Dong, Zhang, Yu, and Wang [29] confirms that a high temperature influences the color change for heat-treated products.

### 3.3. PAH Contamination of Grilled Pork Neck Loins

#### 3.3.1. Influence of Different Grilling Methods on PAH Formation

**Figure 2:**
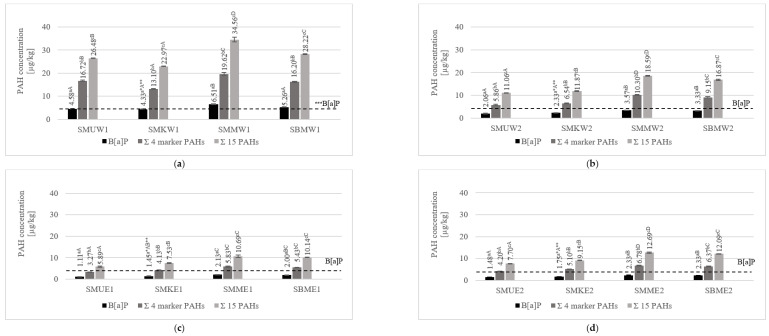
The mean concentration of B[a]P, the sum of 4 marker SCF PAHs, and the sum of 15 SCF PAHs in pork neck loins heat treated using the charcoal grill with no tray (W1), the charcoal grill with an aluminum tray (W2), the ceramic contact grill (E1), and the cast iron grill (E2), and marinated with the use of (**a**) the universal marinade (SMU), (**b**) the pork marinade (SMK), (**c**) the honey mustard marinade (SMM), and (**d**) without marination (SBM). * Within the same grilling method, values for different marination treatments marked with identical lowercase letters (a–c) do not differ significantly at the α = 0.05 level. ** Within the same marination treatment, values for different grills marked with identical capital letters (A–D) do not differ significantly at the α = 0.05 level. *** The horizontal dotted line represents the maximum level for B[a]P specified in Commission Regulation (EU) No. 915/2023 (5 μg/kg).

Figure 2 presents the PAH content in pork neck samples prepared using four grilling tools. The results include the total concentrations of 19 PAHs together with B[a]P, the 4 marker SCF PAHs, and the 15 heavy PAHs. The study’s findings proved the statistical differences in PAH content among the various grilling tools analyzed. Significantly, the highest total contents of 15 polyarenes, 4 marker SCF PAHs, and B[a]P were noted for the pork neck grilled using a charcoal grill with no tray (22.97–34.56 µg/kg, 13.10–19.62 µg/kg, and 4.33–6.51 µg/kg, respectively). The use of an aluminum tray when charcoal grilling significantly reduced the PAH contamination (11.06–18.59 µg/kg, 5.86–10.30 µg/kg, and 2.06–3.57 µg/kg, respectively) by preventing direct contact between the flames and the raw meat. Melting fat from pork neck loins, characterized by a high-fat content, reacted with fire to form PAHs, which, along with the smoke, were was deposited on the surface of the meat. Direct grilling above the heat source without grilling trays means that the PAHs formed in such a process come to a greater extent from fat pyrolysis, and not just from coal combustion. Studies have also shown that samples grilled on electric grills were significantly less contaminated in each variant than those prepared using charcoal grills without a tray, and in most variants from those using charcoal grills with an aluminum tray. Furthermore, it was proved that neck loins grilled on a ceramic electric grill had significantly lower levels of the 15 PAHs and 4 heavy PAHs (5.89–10.69 µg/kg and 3.27–5.83 µg/kg, respectively) than the samples from an electric iron grill (7.70–12.69 µg/kg and 4.20–6.78 µg/kg, respectively). In addition, the B[a]P level for neck loins marinated in the universal marinade also decreased significantly for ceramic contact grills. The differences observed for electric grills are mainly due to the heat treatment temperature and equipment design. The higher cooking temperature contributed to a greater accumulation of harmful compounds. In addition, in the case of the cast iron grill, the lower heating surface was constructed of a corrugated heating plate, where fat deposited in the grooves, thereby promoting a higher level of polyarene formation. In the case of a ceramic grill, the bottom heating surface was flat, and the melting fat did not accumulate in the cavities; therefore, there was no greater accumulation of PAHs.

The obtained results of the grilled neck loin samples’ contamination by PAHs showed that two of the analyzed samples from the charcoal grill with no tray exceeded the limit for B[a]P (SMMW1: 6.51 µg/kg, SBMW1: 5.26 µg/kg); however, they did not exceed the limit for the four heavy PAHs set by the Commission Regulation (EU) No. 915/2023 for heat-treated meat products (respectively, 5 and 30 µg/kg). With these results, it should be recalled that the sum of the four heavy marker PAHs is considered to be a better indicator of the occurrence of PAHs in food than B[a]P alone.

There are many studies aimed at reducing PAH formation in food. One way constantly being researched is to change the grilling method to reduce PAH contamination. In a study by Lee et al. [30], it was observed that when grilling beef loin samples on a charcoal grill with the prevention of fat dripping into the heat source, the amount of harmful B[a]P was significantly lower (0.78 μg/kg) than when fat dripped onto the fire (3.23 μg/kg). In addition, thanks to the use of a modified grill, contamination with the four heavy PAHs decreased by 85%. Moreover, contamination by the four PAHs was 48% lower for pork, which contained less fat. Therefore, this study also confirmed that avoiding direct contact with the fat at the high temperature of the heated flames reduces the formation of PAHs [30]. Fat does not drop into the fire, so PAH formation and deposition onto food surfaces are limited. In addition, preparing meat at high temperatures or long cooking times also leads to a greater formation of PAHs [31]. In the research conducted by Anjum, Shehzad, Rahat, and Khan [32], the type of grilling equipment used significantly affects the safety of grilled products. The study showed that charcoal-grilled meat was more contaminated with PAHs than electric- or gas-grilled meat. It was also confirmed that factors that can prevent the formation of harmful PAHs are lower cooking temperatures and the protection of the food from direct contact with the heat source. Eldaly et al. [33], in a study on the effect of wrapping products in aluminum foil on a reduction in PAH formation, showed that regardless of the meat type, aluminum foil creates a protective barrier between the product and the grill, as a result of which the PAH concentration is significantly lower (beef 1.27 μg/kg and mutton 6.28 μg/kg) compared to the control sample without wrapping (beef 6.83 μg/kg and mutton 26.82 μg/kg). Park, Pyo, Kim, and Yoon [16] found that for traditional grilling, increasing the distance of the product from the heat source and using a barrier to allow air circulation during grilling lowered PAH contamination. Applying these changes reduced the B[a]P concentration by more than six times.

#### 3.3.2. Influence of Different Marination Treatments on PAH Formation

**Figure 3:**
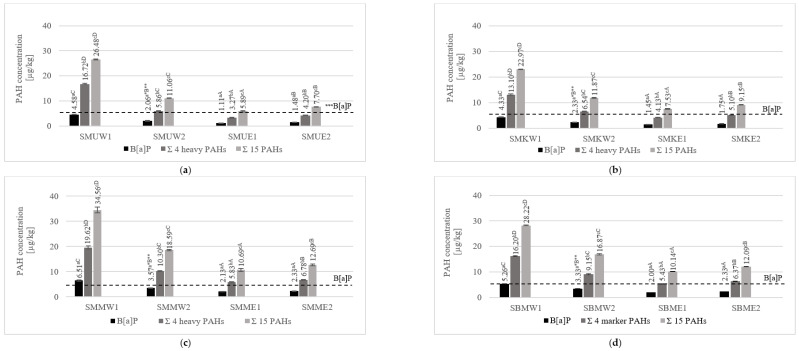
The mean concentration of B[a]P, the sum of 4 marker SCF PAHs, and the sum of 15 PAHs for pork neck loins marinated in universal, pork, and honey mustard marinades and unmarinated (respectively, SMU, SMK, SMM, and SBM) heat treated using (**a**) a charcoal grill with no tray (W1), (**b**) a charcoal grill with an aluminum tray (W2), (**c**) a ceramic contact grill (E1), and (**d**) a cast iron grill (E2). * Within the same marination treatment, values for different grills marked with identical lowercase letters (a–c) do not differ significantly at the α = 0.05 level. ** Within the same grilling method, values for different marination treatments marked with identical capital letters (A–D) do not differ significantly at the α = 0.05 level. *** The horizontal dotted line represents the maximum level for B[a]P specified in Commission Regulation (EU) No. 915/2023 (5 μg/kg).

In preventing PAH contamination, the selected marinades, a kind of green processing technology, were applied to mitigate PAH contamination in grilled pork neck loins. Figure 3 depicts the data of the mean PAH content, including B[a]P, the sum of the 4 marker PAHs, and the 15 SCF PAHs in pork neck loins treated with three different marinades and samples without marination.

The study on whether marination affects PAH contamination in the analyzed products showed that marinades significantly reduce it but, in some cases, can even increase PAH levels in grilled pork on different grilling tools. For the charcoal grill without a tray, the content of the 15 PAHs varied for each of the samples, with, significantly, the lowest concentration (6.31 µg/kg) stated in the pork marinade sample and the highest (8.84 µg/kg) noted for the honey mustard sample. The contents of the four marker PAHs and B[a]P were significantly lower in the universal and pork marinades than in the honey mustard marinade and the samples without marination. On the charcoal grill with a tray, the levels for the sum of the 15 PAHs were also significantly different for each of the variants, with the lowest content in pork prepared with the universal marinade (3.31 µg/kg) and the highest for the honey mustard marinade (5.29 µg/kg). Significantly, the lowest total content for the four heavy marker PAHs was observed in samples treated with universal marinade (1.53 µg/kg) but the highest was for the honey mustard marinade (2.8 µg/kg). Regarding the B[a]P content, significantly, the lowest level of content was stated for the universal marinade, whereas in the other marination treatments, it did not differ significantly. Considering electric grilling, for a ceramic grill with a lower grilling temperature, the lowest significant values for the content of the total 15 PAHs and the 4 marker PAHs were observed in samples with the pork marinade (7.53 µg/kg and 4.13 µg/kg, respectively), and the highest for the honey mustard marination (10.69 µg/kg and 5.83 µg/kg, respectively). In the case of the cast iron grill, statistically the highest total contents of the 15 PAHs and the 4 heavy marker PAHs were found for the honey mustard marinade (12.69 µg/kg and 6.78 µg/kg, respectively) and for pork neck loins with no marination treatment (12.09 µg/kg and 6.37 µg/kg, respectively). Statistically, the lowest concentrations were noted for the pork marinade samples (9.15 µg/kg and 5.10 µg/kg, respectively) and the universal marinade (7.70 µg/kg and 4.20 µg/kg). Furthermore, no statistically significant differences in B[a]P concentrations were observed among the analyzed samples.

The results above confirm that marinating products before their heat treatment can effectively reduce the formation of undesirable contaminants such as PAHs (4 PAHS: 24–29%, 15 PAHs: 31–32%). However, comparing the results to honey mustard-marinated products, it can be concluded that using certain ingredients in marinated products, such as honey, may contribute to increased pollution by PAHs (4 PAHS: 13%, 15 PAHs: 12%) compared to unmarinated necks.

Other researchers also confirm that marination treatment before heat processing can considerably inhibit PAH formation. Cordeiro et al. [34], when preparing pork on a wood grill, showed that marinating in black bean vinegar inhibited the formation of PAHs (82%), as did white wine vinegar (79%). Also, red wine and apple cider vinegar reduced the formation of PAHs by 66%, and the weakest inhibitory effect was shown for fruit vinegar with raspberry juice. Bulanda and Janoszka [35] tested pork tenderloins, which they stuffed with dried fruit and baked in a baking bag. The studies showed that dried fruits significantly reduced contamination with PAHs by 35–58%. Hussein, Edris, and Kirella [36] also noticed that marinating effectively reduced contamination by these carcinogenic compounds. Adding 0.5–1.5% of thyme oil significantly reduced the PAH amount in samples (by 39–74%). Similar results were obtained by Yu et al. [37] in duck wings baked over an open fire and marinated with the addition of coriander. Marination treatment reduced PAH formation from 65.0% to 87.4%. Furthermore, Kim et al. [38], studying the influence of pork belly marination in Korean Gochujang paste, showed a polyarene reduction of 63.06%.

The results obtained in this work have also shown that marination can even contribute to a greater accumulation of polycyclic aromatic hydrocarbons. Comparing the data obtained with the requirements established in Regulation (EU) No. 915/2023 [18] for heat-treated meat products, it was observed that for two samples—unmarinated pork neck loins and those marinated in the honey mustard marinade from the charcoal grill with no tray—the B[a]P limit was exceeded (5.26 µg/kg and 6.51 µg/kg, respectively). Moreover, in this case, the B[a]P concentration increased due to marinating with this marinade by about 24% compared to the unmarinated pork neck loins.

Honey used for marinating is an ingredient susceptible to pollution by polyarenes. Bees taking long flights visit many areas contaminated by harmful elements from the soil, air, and other environmental pollutants such as PAHs [21]. Kazazic, Djapo-Lavic, Mehic, and Jesenkovic-Habul [39] studied PAH contamination in honey from urbanized and non-urbanized areas. The level of total PAH concentration in honey taken from industrial areas was almost five times higher (12.58 μg/kg) compared to beehives situated far from cities and factories (2.68–4.76 μg/kg). Significant differences in the polyarene contents of honey depending on the region of origin are also concluded in the research by Surma, Sadowska-Rociek, and Draszanowska [40]. In addition, the harmful effect of honey on PAH formation is also influenced by the high temperature during the grilling process, which changes in a manner associated with the Maillard reaction to cause the formation of PAHs. The harmful effects of honey added to marinades were also investigated by Nor Hasyimah et al. [41] by grilling marinated beef over a fire. The studies reported that the samples previously treated with a spiced honey marinade and then prepared on the grill had a significantly higher concentration of PAHs (B[a]P: 4.49–32.60 μg/kg and 8 PAHs: 78.47–164.40 μg/kg) compared to unmarinated samples (B[a]P 2.67–4.60 μg/kg and 8 PAHs: 34.59–89.52 μg/kg). Furthermore, the researchers formulated the conclusion that samples after marination in *Apis mellifera* honey were characterized by lower total PAH contamination compared to those treated with honey from the honeybee *Trigona*. These differences were due to the content of simple carbohydrates, which, under the influence of high temperatures, degrade to chloropropanols, which are precursors in PAH formation. There were significantly fewer reducing sugars in *Apis mellifera* honey than in *Trigona* bee honey.

#### 3.3.3. Qualitative PAH Contamination

**Figure 4:**
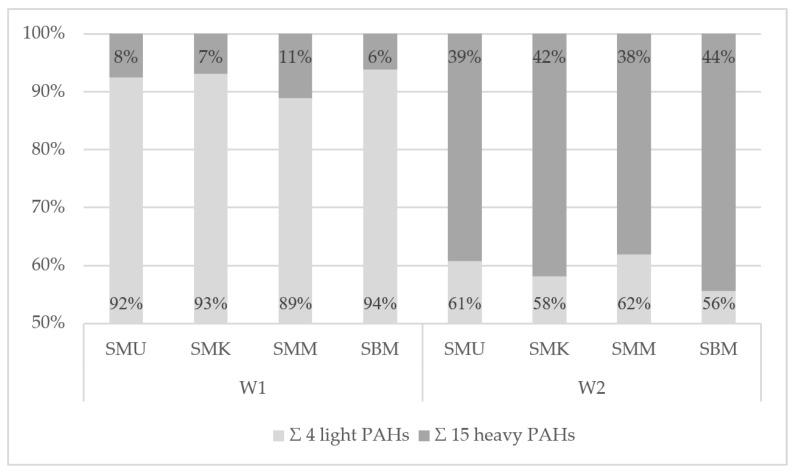
Percentage share of the sum of 4 light and 15 heavy marker PAHs in the contamination profile of pork neck loins from the charcoal grill with no tray (W1) and the charcoal grill with an aluminum tray (W2).

**Figure 5:**
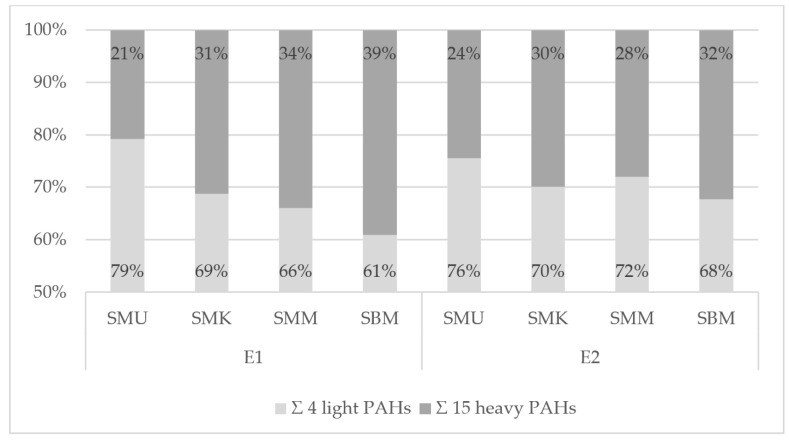
Percentage share of the sum of 4 light and 15 heavy-marker PAHs in the contamination profile of pork neck loins from the electric ceramic contact grill (E1) and the electric cast iron contact grill (E2).

Profiles of the pork neck samples’ qualitative PAH contamination were analyzed with a percentage of 4 light and 15 heavy SCF PAHs (Figure 4, Table S1). For meat heat-treated with the charcoal grill with no tray, a higher level of light hydrocarbons (W1: 89–94%) was recorded than for the samples prepared on an aluminum tray (W2: 56–62%). The pyrolysis of the fats during the grilling of the samples was the main factor for which the values between these samples differed. The fat melting from the pork neck loins and falling onto the heated coals led to the formation of PAHs, which, together with the smoke, were lifted upwards and then deposited onto the surface of the products. The aluminum tray effectively reduced flame-to-fat contact, resulting in a lower proportion of formed light hydrocarbons. For electric grills (Figure 5, Table S2), the PAH contamination profiles did not reveal such a differentiation between the ceramic and cast iron grills (E1: 61–79%, E2: 68–76%). When analyzing the different variants of the samples on the grills applied, there were differences between the light and heavy PAHs. The type of device, its design, the grilling parameters, and the previous preparation of the raw material are important factors affecting grilled meat product PAH contamination [12].

## 4. Conclusions

The consumption of grilled meat products can threaten human health and sometimes even exceed permitted daily intake norms. Subjecting pork neck loins to grilling on various grilling devices after their marinating proved that a conscious and proper choice of marinades, like the universal or pork marinade applied in this study, and constituting a rich source of phenols, as well as a conscious choice of a proper grilling device, like electric grills over charcoal grills, can significantly improve food safety. Charcoal grills, due to the direct exposure of the raw material to the heat source, result in the greatest PAH concentrations. An aluminum tray effectively reduces the amount of accumulated PAHs, as does using electric grills. In addition, marination also significantly affects the amount of PAHs. Therefore, key strategies for minimizing PAH concentrations in green processing technology involve marination before heat treatment. Marinades rich in phenolic compounds allow for the reduction in PAH amounts and ensure product safety. However, our research on the marinades that are popular in Poland, the use of which for pork loins has not been previously tested for PAH contamination, showed that marinating can also increase PAH contamination in grilled meat products. Therefore, the results obtained in this work direct attention to the marinade ingredients, which can cause greater contamination by polyarenes. The honey used for marinades caused higher PAH concentrations and, in some samples, even exceeded the permitted B[a]P level. It is imperative to conduct further research investigating the influence of various marinades and grills and expand knowledge on reducing PAH contamination in processed meat products to develop effective strategies for the minimization of the dietary intake of these heat-induced toxicants. These measures are necessary to increase food safety and safeguard public health.

## Figures and Tables

**Table 1:** Compositional profiles of marinades.

Marinade Type	Ingredients [g/100 g of Meat]	Code
Universal marinade	5 g of universal seasoning (composition: salt, rosemary (10.3%), basil, sugar, onion, paprika, oregano, marjoram, ground mustard, coriander, sunflower oil, thyme, lemon juice, turmeric, and cayenne pepper), of 5 g refined rapeseed oil	MU
Pork marinade	5 g of pork seasoning (composition: salt, garlic (22.0%), red pepper (14.0%), coriander, rosemary, black pepper, citric acid, parsley (2.2%), and bay leaves), 5 g of refined rapeseed oil	MK
Honey mustard marinade	5 g of spicy mustard (water, white mustard, spirit vinegar, black mustard, sugar, salt, aroma, and turmeric extract), 3.5 g of lime honey, 1 g of refined rapeseed oil, 0.5 g of freshly squeezed lemon juice	MM

**Table 2:** Parameters of grilling.

Type of Grill	Grill Temperature [°C]	Grilling Time [min]	Temperature at the End of the Grilling Process in the Product’s Geometric Center [°C]
W1	240–300	12	94.5
W2	170–220	17	90.2
E1	180–200	15	93.2
E2	200–220	15	94.9

W1—charcoal grill with no tray, W2—charcoal grill with an aluminum tray, E1—electric ceramic contact grill, E2—electric cast iron contact grill.

**Table 3:** Weight loss of pork neck loins, subjected to different marination treatments, after grilling.

Universal Marinade		Pork Marinade		Honey Mustard Marinade		Without Marinade	
Sample Code	Weight Loss [%]	Sample Code	Weight Loss [%]	Sample Code	Weight Loss [%]	Sample Code	Weight Loss [%]
SMUW1	41.1 ± 1.63 ^c^*	SMKW1	54.7 ± 0.57 ^d^	SMMW1	45.4 ± 0.32 ^b^	SBMW1	50.3 ± 0.23 ^c^
SMUW2	30.0 ± 0.00 ^a^	SMKW2	32.0 ± 0.50 ^a^	SMMW2	43.4 ± 0.61 ^a^	SBMW2	34.5 ± 0.62 ^a^
SMUE1	37.2 ± 0.05 ^b^	SMKE1	38.0 ± 0.12 ^b^	SMME1	52.0 ± 0.08 ^c^	SBME1	45.9 ± 0.09 ^b^
SMUE2	46.4 ± 0.05 ^d^	SMKE2	43.4 ± 0.13 ^c^	SMME2	53.5 ± 0.15 ^d^	SBME2	54.8 ± 0.00 ^d^

n = 6. * Within the same marination treatment, values marked with identical lowercase letters (a–d) do not differ significantly in a given comparison, as determined at the α = 0.05 level. S—pork neck loins, MU—universal marinade, MK—pork marinade, MM—honey mustard marinade, BM—with no marinade. W1—charcoal grill with no tray, W2—charcoal grill with an aluminum tray, E1—ceramic contact grill, E2—cast iron contact grill.

**Table 4:** Color of pork neck loins subjected to different marination treatments before and after heat treatment.

Grilling Method	Sample	L*	a*	b*
Before grilling	SMU	33.66 ± 0.75 ^a^*^A^**	10.52 ± 0.92 ^aB^	12.66 ± 1.77 ^bA^
	SMK	37.16 ± 0.28 ^aA^	10.99 ± 0.83 ^aB^	5.38 ± 0.61 ^abA^
	SMM	52.19 ± 0.56 ^bB^	11.66 ± 1.55 ^aA^	8.01 ± 0.86 ^bA^
	SBM	44.87 ± 2.90 ^bB^	13.16 ± 1.05 ^aC^	4.24 ± 0.42 ^aA^
Charcoal grill without a tray	SMUW1	38.38 ± 2.43 ^aA^	6.54 ± 0.27 ^aA^	14.01 ± 1.09 ^bB^
	SMKW1	39.18 ± 4.29 ^aA^	9.00 ± 2.15 ^aB^	7.46 ± 0.77 ^aA^
	SMMW1	48.46 ± 1.67 ^bAB^	9.25 ± 0.87 ^aA^	10.55 ± 0.61 ^abA^
	SBMW1	44.70 ± 3.80 ^bA^	12.21 ± 2.16 ^bBC^	7.91 ± 3.04 ^aAB^
Charcoal grill with an aluminum tray	SMUW2	40.89 ± 2.11 ^aA^	6.32 ± 1.25 ^aA^	14.45 ± 1.00 ^bB^
	SMKW2	46.42 ± 1.22 ^aB^	5.25 ± 0.63 ^aA^	12.38 ± 1.25 ^abB^
	SMMW2	44.30 ± 3.96 ^aA^	11.01 ± 0.31 ^bA^	10.45 ± 0.26 ^aA^
	SBMW2	55.88 ± 2.20 ^bC^	7.82 ± 0.67 ^aA^	11.31 ± 1.32 ^abBC^
Ceramic contact grill	SMUE1	40.59 ± 4.92 ^aA^	6.86 ± 0.85 ^aA^	8.59 ± 1.22 ^aA^
	SMKE1	40.26 ± 1.40 ^aAB^	8.03 ± 1.00 ^aAB^	7.52 ± 1.73 ^aA^
	SMME1	49.45 ± 2.73 ^bAB^	8.71 ± 0.65 ^aA^	9.84 ± 0.76 ^abA^
	SBME1	47.28 ± 2.49 ^bB^	8.91 ± 0.94 ^aA^	13.51 ± 1.74 ^bC^
Cast iron contact grill	SMUE2	35.02 ± 1.26 ^aA^	7.69 ± 0.80 ^aA^	10.82 ± 0.92 ^abAB^
	SMKE2	40.24 ± 1.62 ^aAB^	8.42 ± 2.18 ^aB^	7.71 ± 1.59 ^aA^
	SMME2	46.87 ± 0.68 ^bAB^	8.25 ± 0.25 ^aA^	9.39 ± 0.33 ^abA^
	SBME2	46.96 ± 4.86 ^bB^	9.85 ± 1.89 ^aAB^	11.76 ± 3.40 ^bC^

n = 6. * Within the same grilling method, values marked with identical lowercase letters (a,b) do not differ significantly at the α = 0.05 level. ** Within the same marination treatment, values marked with identical capital letters (A–C) do not differ significantly at the α = 0.05 level.

**Table 5:** The color differences for pork neck loins before and after heat treatment.

Charcoal Grill with No Tray		Charcoal Grill with an Aluminum Tray		Ceramic Contact Grill		Cast Iron Contact Grill	
Sample	ΔE	Sample	ΔE	Sample	ΔE	Sample	ΔE
SMUW1	6.32 ± 2.59 ^a^*^A^**	SMUW2	8.55 ± 2.66 ^abA^	SMUE1	14.96 ± 4.45 ^bB^	SMUE2	4.53 ± 0.85 ^aA^
SMKW1	3.52 ± 1.22 ^aA^	SMKW2	12.95 ± 1.54 ^bB^	SMKE1	4.64 ± 1.74 ^aA^	SMKE2	5.54 ± 1.08 ^aA^
SMMW1	5.12 ± 1.81 ^aA^	SMMW2	8.29 ± 3.07 ^aA^	SMME1	4.42 ± 1.95 ^aA^	SMME2	6.46 ± 1.05 ^aA^
SBMW1	14.51 ± 2.36 ^bA^	SBMW2	11.40 ± 3.18 ^bA^	SBME1	10.30 ± 1.37 ^bA^	SBME2	12.51 ± 1.95 ^bA^

* Within the same grilling method, values marked with identical lowercase letters (a,b) do not differ significantly at the α = 0.05 level. ** Within the same marination treatment, values marked with identical capital letters (A,B) do not differ significantly at the α = 0.05 level.

## Data Availability

The original contributions presented in the study are included in the article/Supplementary Materials, and further inquiries can be directed to the corresponding author.

## Supplementary Materials

The following supporting information can be downloaded at: https://www.mdpi.com/article/10.3390/foods14101673/s1, Table S1: Individual PAH concentrations in pork neck loins after marination treatment from the charcoal grill with no tray (W1) and with an aluminum tray (W2); Table S2: Individual PAH concentrations in pork neck loins after marination treatment from the electric ceramic contact grill (E1) and the cast iron contact grill (E2).
