# Supplementary material for: Formation of Polycyclic Aromatic Hydrocarbons on Grilled Pork Neck Loins as Affected by Different Marinades and Grill Types
Source: Foods. 2025 May 9;14(10):1673. doi: 10.3390/foods14101673 (PMC12111158; doi:10.3390/foods14101673)
Supplement: Supplementary file 1 [file foods-14-01673-s001.zip › foods-3607508-supplementary.pdf]

Table S1. Individual PAH concentrations in pork neck loins after marination treatment from the charcoal grill with no tray (W1) and with an aluminum tray (W2).

PAH	PAH concentration [µg/kg]							
	**SMUW1**	**SMKW1**	**SMMW1**	**SBMW1**	**SMUW2**	**SMKW2**	**SMMW2**	**SBMW2**
Phen	188.65±8.35	220.27±15.04	171.04±6.38	27.39±12.18	8.69±0.11	10.24±0.70	17.25±0.55	11.40±0.60
Anthr	12.04±0.53	14.06±0.96	10.92±0.41	17.45±0.78	1.36±0.29	0.54±0.02	2.49±0.23	1.37±0.07
F	101.97±7.08	60.69±4.23	76.60±3.92	108.96±4.91	5.46±0.02	3.15±0.28	8.13±0.69	6.08±0.45
Pyr	21.26±1.37	16.82±0.07	18.36±0.70	24.07±1.16	1.59±0.12	2.56±0.03	2.36±0.04	2.23±0.02
C[cd]P	nd[x]	nd	nd	nd	nd	nd	nd	nd
B[a]A	5.48±0.18	2.82±0.11	5.09±0.15	3.74±0.36	1.27±0.03	1.34±0.02	2.00±0.0	1.95±0.06
Chr	3.98±0.05	3.31±0.02	4.25±0.12	3.80±0.09	1.30±0.01	1.41±0.01	2.62±0.14	1.95±0.06
5-MChr	nd	nd	nd	nd	nd	nd	nd	nd
B[j]F	nd	nd	nd	nd	nd	nd	nd	nd
B[b]F	2.67±0.01	2.64±0.06	3.77±0.15	3.11±0.05	1.23±0.02	1.45±0.12	2.10±0.01	1.93±0.06
B[k]F	2.34±0.02	2.36±0.07	3.59±0.11	2.93±0.04	1.23±0.02	1.33±0.01	1.99±0.01	1.88±0.06
B[a]P	4.58±0.04	4.33±0.09	6.51±0.25	5.26±0.10	2.06±0.12	2.33±0.03	3.57±0.04	3.33±0.11
D[ah]A	nd	nd	nd	nd	nd	nd	nd	nd
D[al]P	2.42±0.03	2.44±0.19	3.56±0.12	2.92±0.04	1.29±0.01	1.38±0.01	1.97±0.01	1.88±0.05
B[ghi]P	2.91±0.16	2.84±0.14	4.40±0.06	3.50±0.24	1.64±0.16	1.59±0.06	2.48±0.21	2.30±0.17
I[cd]P	2.10±0.02	2.22±0.09	3.38±0.23	2.66±0.14	1.04±0.05	1.04±0.08	1.85±0.05	1.64±0.07
D[ae]P	nd	nd	nd	nd	nd	nd	nd	nd
D[ai]P	nd	nd	nd	nd	nd	nd	nd	nd
D[ah]P	nd	nd	nd	nd	nd	nd	nd	nd

n = 6 (six samples of every kind of product were analyzed). [x] nd – not detected. S – pork neck loins. MU – universal marinade, MK – pork marinade, MM – honey mustard marinade, BM – without marinade. W1 – charcoal grill without a tray, W2 – charcoal grill with an aluminum tray.

Table S2. Individual PAH concentrations in pork neck loins after marination treatment from the electric ceramic contact grill (E1) and cast iron contact grill (E2).

PAH	PAH concentration [µg/kg]							
	SMUE1	SMKE1	SMME1	SBME1	SMUE2	SMKE2	SMME2	SBME2
Phen	12.19±0.35	8.19±0.18	9.06±0.58	9.60±0.59	9.46±0.23	11.58±0.55	17.39±1.77	14.55±1.30
Anthr	1.26±0.13	0.51±0.03	0.76±0.08	0.58±0.06	1.89±0.13	0.52±0.05	0.89±0.04	0.71±0.07
F	7.46±0.53	5.97±0.10	8.75±0.95	3.51±0.08	10.73±0.43	6.36±0.00	10.90±1.63	7.52±0.26
Pyr	1.49±0.13	1.93±0.04	2.17±0.03	2.09±0.14	1.73±0.12	2.97±0.07	3.51±0.12	2.55±0.22
C[cd]P	nd[x]	nd	nd	nd	nd	nd	nd	nd
B[a]A	0.78±0.10	0.92±0.02	1.25±0.07	1.16±0.00	0.93±0.03	1.06±0.02	1.47±0.05	1.36±0.03
Chr	0.70±0.02	0.89±0.05	1.21±0.03	1.12±0.02	0.88±0.06	1.05±0.02	1.43±0.03	1.32±0.03
5-MChr	nd	nd	nd	nd	nd	nd	nd	nd
B[j]F	nd	nd	nd	nd	nd	nd	nd	nd
B[b]F	0.68±0.04	0.88±0.02	1.24±0.05	1.14±0.00	0.91±0.00	1.24±0.06	1.56±0.05	1.37±0.03
B[k]F	0.58±0.02	0.80±0.02	1.18±0.06	1.10±0.00	0.82±0.01	0.97±0.01	1.45±0.03	1.30±0.03
B[a]P	1.11±0.03	1.45±0.03	2.13±0.11	2.00±0.01	1.48±0.01	1.75±0.02	2.33±0.08	2.33±0.04
D[ah]A	nd	nd	nd	nd	nd	nd	nd	nd
D[al]P	0.69±0.07	0.86±0.02	1.20±0.08	1.11±0.03	0.87±0.00	1.01±0.01	1.45±0.05	1.33±0.03
B[ghi]P	0.75±0.08	0.94±0.03	1.35±0.08	1.52±0.21	0.98±0.01	1.11±0.01	1.63±0.04	1.83±0.20
I[cd]P	0.60±0.02	0.80±0.05	1.14±0.11	0.98±0.03	0.83±0.03	0.97±0.02	1.38±0.05	1.26±0.13
D[ae]P	nd	nd	nd	nd	nd	nd	nd	nd
D[ai]P	nd	nd	nd	nd	nd	nd	nd	nd
D[ah]P	nd	nd	nd	nd	nd	nd	nd	nd

n = 6 (six samples of every kind of product were analyzed). [x] nd – not detected. S – pork neck loins. MU – universal marinade, MK – pork marinade, MM – honey mustard marinade, BM – without marinade. E1 – ceramic contact grill, E2 – cast iron contact grill.
